# Somatic Copy Number Abnormalities and Mutations in PI3K/AKT/mTOR Pathway Have Prognostic Significance for Overall Survival in Platinum Treated Locally Advanced or Metastatic Urothelial Tumors

**DOI:** 10.1371/journal.pone.0124711

**Published:** 2015-06-03

**Authors:** Joaquim Bellmunt, Lillian Werner, Jeffrey J. Leow, Stephanie A. Mullane, André P. Fay, Markus Riester, Paul Van Hummelen, Mary-Ellen Taplin, Toni K. Choueiri, Eliezer Van Allen, Jonathan Rosenberg

**Affiliations:** 1 Lank Center for Genitourinary Oncology, Dana-Farber Cancer Institute/Brigham and Women's Hospital, Harvard Medical School, Boston, MA, United States of America; 2 Biostatistics and Computational Biology, Harvard Medical School, Dana-Farber Cancer Institute, Boston, MA, United States of America; 3 Center for Cancer Genome Discovery, Dana-Farber Cancer Institute, Boston, MA, United States of America; 4 Memorial Sloan Kettering Cancer Center, New York, NY, United States of America; Istituto dei tumori Fondazione Pascale, ITALY

## Abstract

**Background:**

An integrative analysis was conducted to identify genomic alterations at a pathway level that could predict overall survival (OS) in patients with advanced urothelial carcinoma (UC) treated with platinum-based chemotherapy.

**Patients and Methods:**

DNA and RNA were extracted from 103 formalin-fixed paraffin embedded (FFPE) invasive high-grade UC samples and were screened for mutations, copy number variation (CNV) and gene expression analysis. Clinical data were available from 85 cases. Mutations were analyzed by mass-spectrometry based on genotyping platform (Oncomap 3) and genomic imbalances were detected by comparative genomic hybridization (CGH) analysis. Regions with threshold of log^2^ ratio ≥0.4, or ≤0.6 were defined as either having copy number gain or loss and significantly recurrent CNV across the set of samples were determined using a GISTIC analysis. Expression analysis on selected relevant UC genes was conducted using Nanostring. To define the co-occurrence pattern of mutations and CNV, we grouped genomic events into 5 core signal transduction pathways: 1) TP53 pathway, 2) RTK/RAS/RAF pathway, 3) PI3K/AKT/mTOR pathway, 4) WNT/CTNNB1, 5) RB1 pathway. Cox regression was used to assess pathways abnormalities with survival outcomes.

**Results:**

35 samples (41%) harbored mutations on at least one gene: *TP53* (16%), *PIK3CA* (9%), *FGFR3* (2%), *HRAS/KRAS* (5%), and *CTNNB1* (1%). 66% of patients had some sort of CNV. PIK3CA/AKT/mTOR pathway alteration (mutations+CNV) had the greatest impact on OS (p=0.055). At a gene level, overexpression of *CTNNB1* (p=0.0008) and *PIK3CA* (p=0.02) were associated with shorter OS. Mutational status on *PIK3CA* was not associated with survival. Among other individually found genomic alterations, *TP53* mutations (p=0.07), *mTOR* gain (p=0.07) and *PTEN* overexpression (p=0.08) have a marginally significant negative impact on OS.

**Conclusions:**

Our study suggests that targeted therapies focusing on the PIK3CA/AKT/mTOR pathway genomic alterations can generate the greatest impact in the overall patient population of high-grade advanced UC.

## Introduction

Urothelial carcinoma (UC) is a common malignancy in the United States with nearly 75,000 cases diagnosed annually, and with more than 15,000 disease-related deaths[1]. UC is categorized into non-muscle invasive bladder cancer (NMIBC) and muscle-invasive bladder cancer (MIBC). About 70% of UC tumors present as NMIBC, which is usually low grade and indolent[2]. MIBC consists of the other 30% of UC and generally portends a poor prognosis, with 5-year overall survival rates of 50% when treated with neoadjuvant chemotherapy plus radical cystectomy[3].

NMIBC and MIBC have been described as heterogeneous tumors with different genomic landscapes. NMIBC is generally characterized by mutations in *FGFR3*, *RAS*, and deletions on chromosome 9, while MIBC is characterized by mutations in *TP53*, *RB1*, *PIK3CA* and *PTEN*[4–6]. The concept that most malignancies depend on driver mutations to establish and maintain the malignant phenotype has been established, although involvement of other genomic events and epigenetic alterations has been also recognized as potential drivers for carcinogenesis.

The Cancer Genome Atlas (TCGA) Research Network recently published a comprehensive molecular characterization of urothelial bladder carcinoma and recurrent mutations in 32 genes, including *TP53*, *FGFR3*, *ERCC2*, *NF2*, and *CDKN1A* were described to play a role in UC tumorigenesis[7]. Limited data are available from this analysis associating single or grouped genomic alterations with survival outcomes in patients with UC.

In this study, we present an integrative analysis of multiple types of genomic data, including mutations, copy number variations (CNV), and messenger RNA (mRNA) expression data from 103 metastatic UC patients treated at two institutions with first-line platinum-based combination chemotherapy. We have combined genomic events on 5 signal transduction pathways known to be important in UC tumorigenesis and maintenance of a malignant phenotype, and correlated these findings with clinical outcomes. The 5 cancer signaling pathways selected for this analysis were TP53, RTK/RAS/ /RAF, PI3KCA/AKT/mTOR, WNT/CTNNB1, and RB1.

## Material and Methods

### Patients

This project was approved by the local ethics committee (CEIC-IMAS) at Hospital del Mar and by the Dana-Farber/Harvard Cancer Center (DF/HCC) Institutional Review Board (IRB). A total of 103 clinically annotated patients with locally advanced or metastatic UC who were treated with first-line platinum-based combination chemotherapy were identified and tumor specimens were retrieved from the Pathology Departments. Since the majority of patients were dead at the time of sample collection, a waiver of consent was requested and given from the DF/HCC for all participants (requiring complete de-identification of the samples prior the analysis).

### Tumor Samples

DNA was extracted from formalin-fixed paraffin embedded (FFPE) material using the QIAamp DNA FFPE Tissue Kit (Qiagen, Valencia, CA). We performed comparative genomic hybridization (CGH) to determine genomic imbalances using genomic DNA isolated from primary tumors as well as karyotypically normal reference genomic DNA (Promega, Madison, WI). Agilent Oligonucleotide Human Genome 180k CGH arrays were used to perform the analysis. The Genomic DNA ULS labeling kit for FFPE Samples (Agilent Technologies, Inc., Palo Alto, CA) was used to chemically label 500ng of DNA with either ULS-Cy5 (tumor) or ULS-Cy3 dye (normal/reference DNA) following the manufacturer's protocol. Samples were hybridized to the Agilent SurePrint G3 Human CGH Microarray 4x180K for 40 hours in a Robbins Scientific oven with rotation at 20 rpm at 65°C. Post-hybridization, the slides were washed and scanned using an Agilent DNA microarray scanner. CGH Analytics software (version 3.4, Agilent Technologies, CA) was used to evaluate the aCGH data. The GISTIC module was used to identify regions of the genome that were lost or gained across the set of samples. Regions with threshold of log2 ratio of ≥0.4, or ≤ -0.3 were defined as either having copy number gain or loss, respectively. Throughput mutation profiling was performed by using both mass spectroscopy-based genotyping (Oncomap 3 platform) and confirmed with hME sequencing.

mRNA expression profile was obtained by using Nanostring technology. mRNA was extracted from tumor specimens using standard protocols. Oligonucleotide probes for all genes analyzed were synthesized by Nanostring Technologies, and transcripts were counted using the automated Nanostring nCounter system. Counts were normalized with the nSolver Analysis Software (v1.0) in which mRNA expression was compared to internal Nanostring controls, several housekeeping (ACTB, GAPHD, HPRT1, LDHA, PFKP, PGAM1, STAT1, TUBA4A, VIM) and invariant genes in bladder cancer (ANGEL1, DDX19A, NAGA, RPS10, RPS16, RPS24, RPS29). These invariant genes were identified by analyzing gene expression variances in several published datasets (12, 14, 15-1q23).

### Statistical Analysis

Overall survival (OS) was defined as the time period from the first chemotherapy administration to the date of death or censored on the last known alive date. Median OS rates were calculated based on date of death or last known alive date. Using the log-rank test and univariate Cox proportional regression, we tested the primary endpoint of this study which was to evaluate the association between pathway abnormalities (mutations, CNV, both genomic alterations taken together, and expression analysis in signaling pathways) and OS. For 85 patients with mutational and clinical data, mutational status of each gene identified was examined in multivariate Cox regression models. The association of CNV status (gain/amplification vs. loss) and OS was evaluated in 93 patients with CNV and clinical data available. Additionally, for the 74 patients having clinical and expression data from nanostring, we examined the association of overexpression of selected genes within a pathway with OS, adjusting for clinical variables (performance status and visceral metastases).

All statistical analyses were performed using SAS 9.3 (SAS Institute, NC). All tests were two-sided and a p-value of <0.05 was considered statistically significant.

## Results

A total of 103 metastatic UC patients treated with first-line platinum-based combination chemotherapy were included in the analysis. Clinical characteristics are outlined in Table 1. Median follow up was 23 months. The median OS since the application of first chemotherapy for treatment of metastatic disease was 12 months.

**Table 1:** Patients and Clinical Characteristics.

	N	% or median (q1,q3)
ECOG PS		
0	37	35%
1, 2	66	64%
Visceral disease		
Yes	37	36%
No	66	64%
Pathological stage		
Stage 0 (Ta)	10	10%
Stage I (T1)	5	5%
Stage II (T2)	50	49%
Stage III (T3, T4)	31	30%
Stage IV (L, M)	5	5%
Missing	2	2%
Hemoglobin	100	12.7 (8.8, 15.5)
Missing	3	3%

### Association of Mutational Status and Survival Outcome

Clinical data were available for 85 out of 93 samples (91%) scanned for gene mutations and those 85 were included in the analysis. Using the Oncomap 3 platform, we identified the proportion of patients with gene mutations: *TP53* (16%), *PIK3CA* (11%), *HRAS/KRAS* (5%), *FGFR3* (2%), *CTNNB1* (1%), *BRAF* (1%) and *ERBB2* (1%) (Table 2).

**Table 2:** Name of Gene & Candidate Mutation validated by hME.

Name of Gene & Candidate Mutation	
Gene	Mutation
*BRAF*	G466A
*CTNNB1*	N287S
*ERBB2*	L755S
*ERBB2*	V777L
*FGFR3*	R248C
*FGFR3*	Y373C
*HRAS*	G13R
*HRAS*	G12S
*KRAS*	G12D
*PIK3CA*	E545K
*PIK3CA*	H1047R
*TP53*	R175H
*TP53*	R248W
*TP53*	R248Q
*TP53*	R273C
*TP53*	R273H
*TP53*	E285K
*TP53*	R213*
*TP53*	V157F
*TP53*	Y220C

We examined whether the most frequent 3 mutations above were associated with OS. Due to the small mutation prevalence in the other genes detected with this platform (0–2%), those were not included in the analysis. *TP53* mutation was associated with a shorter median OS (10 vs. 16 months) trending toward significance (p = 0.07). Mutations of *PIK3CA* (not reached vs. 14 months, p = 0.26) and *HRAS/KRAS* (17 vs. 12 months, p = 0.17) alone were not associated with shorter OS (Table 3). All other mutations were not significantly associated with OS.

**Table 3:** Mutation status of genes.

	N	%
***PIK3CA***		
Unmutated	76	89%
Mutated	9	11%
***FGFR3***		
Unmutated	83	98%
Mutated	2	2%
***HRAS/KRAS***		
Unmutated	81	95%
Mutated	4	5%
***CTNNB1***		
Unmutated	84	99%
Mutated	1	1%
***ERBB2***		
Unmutated	85	100%
Mutated	0	0%
***TP53***		
Unmutated	71	84%
Mutated	14	16%
***BRAF***		
Unmutated	84	99%
Mutated	1	1%

### Association of CNV and Survival Outcome

CNV were identified in 66% of patients. Using the GISTIC algorithm[8] we identified 96 focal (< 50% of a chromosome arm) and 22 broad (> 50% of a chromosome arm) CNV events. Association between CNV and OS were evaluated in 94 patients with corresponding clinical data available. Adverse genomic alterations were reported according to the respective signal transduction pathway: (1) TP53 (*TP53* loss 13%), (2) RTK/RAS/RAF (*RAF1* gain 24%, *ERBB2* gain 19%, *FGFR1* gain 12%, *BRAF* gain 5%, *KRAS* gain 4%, *MET* gain 4%, *FGFR3* gain 2%, *NF1* loss 1%); (3) PI3K/ATK/mTOR (*TSC1* loss 13%, *PIK3CA* gain 10%, *PTEN* loss 6%, *AKT1* gain 2%, *MTOR* gain 2%), (4) WNT/CTNNB1 (CTNNB1 gain 4%) and (5) RB1 (*CCND1* gain 11%, *CCNE1* gain15, *CDKN2A* loss 2%, *E2F3* gain 28%, *RB1* gain 88%). None of the CNV events alone were significantly correlated with OS **(** Table 4). The only individual CNV which correlated with a trend in terms of OS benefit was a copy number gain of *mTOR* (p = 0.07).

**Table 4:** Association of CNV of genes In TP53, RTK/RAS/RAF, PI3KCA/AKT/mTOR, WNT/CTNNB1 and RB1 pathways with OS.

CNV (Log2 ratio)	N	Death	Median OS	p-value
***RTK/RAS/RAF pathway***				
ERBB2				0.45
<0.4	76	35	15	
≥0.4	18	11	14	
MET				0.59
<0.4	90	44	14	
≥0.4	4	2	Not reached	
FGFR1				0.72
<0.4	82	40	15	
≥0.4	12	6	23	
KRAS				0.52
<0.4	90	45	14	
≥0.4	4	1	Not reached	
BRAF				0.64
<0.4	89	44	15	
≥0.4	5	2	Not reached	
RAF1				0.29
<0.4	72	37	14	
≥0.4	22	9	Not reached	
***PI3K/AKT/mTOR pathway***				
PTEN				0.14
>-0.3	88	42	16	
≤-0.3	6	4	9	
PIK3CA				0.14
<0.4	85	39	16	
≥0.4	9	7	12	
AKT1				0.26
<0.4	92	46	14	
≥0.4	2	0	Not reached	
TSC1				0.14
>-0.3	82	38	18	
≤-0.3	12	8	11	
MTOR				0.07
<0.4	92	44	16	
≥0.4	2	2	7	
***TP53 pathway***				
TP53 loss				0.86
>-0.3	82	39	16	
≤-0.3	12	7	14	
**WNT/CTNNB1 *pathway***				
CTNNB1 gain				0.67
<0.4	90	44	14	
≥0.4	4	2	Not reached	
**RB1 pathway**				
CCND1 gain				0.14
<0.4	83	39	18	
≥0.4	11	7	11	
CCNE1 gain				0.81
<0.4	80	39	15	
≥0.4	14	7	18	
CDN2A loss				-
>-0.3	92	46	15	
≤-0.3	2	0	Not reached	
E2F3 gain				0.63
<0.4	68	31	15	
≥0.4	26	15	16	
RB1 gain				0.57
>-0.3	83	40	15	
≤-0.3	11	6	16	

Note: CDK4 and CDN2A were not analyzed due to small number of patients with copy number variation.

Focal gains around *ERBB2* were correlated with an increase *ERBB2* mRNA expression as compared with non-amplified samples. However, neither *ERBB2* mRNA overexpression nor CNV (gain) were associated with survival outcomes. We also observed a slight increase in mRNA expression of *RAF1* in samples with copy number gain. There were no changes in mRNA expression in patients with CNV in *BRAF*, *FGFR3*, and *KRAS* locus.

### Integrative Pathway Analysis and Correlation with Clinical Outcome

Patients with genomic events (CNV, mutation, and gene expression) in the TP53, RTK/RAS/RAF, PI3K/AKT/mTOR, WTN/CTNNB1, or RB1 signaling pathways were analyzed according to survival outcomes.

#### TP53 pathway

The *MDM2* gene, which is part of the TP53 pathway, was evaluated for mutations, CNV and gene expression and no genomic events were identified. Therefore, genomic events in this pathway are represented by *TP53* gene mutation. TP53 mutation was correlated with a shorter OS (p = 0.07) (S1 Table).

#### RTK/RAS/RAF pathway

The association of mutational status and CNV of all genes within the RTK/RAS/RAF pathway [*ERBB2* (copy number gain or mutation), *FGFR3* (copy number gain or mutation), *MET* (copy number gain), *FGFR1* (copy number gain), *KRAS* (copy number gain or mutation), *HRAS* (mutation), *BRAF* (copy number gain or mutation), *RAF1* (copy number gain), *NF1* (copy number loss)] with OS was not statistically significant (p = 0.56) (S1 Table). Genomic events within the RTK/RAS/RAF pathway were mutually exclusive (data not shown). There were overlaps of RAF1 and FGFR3 genomic events in 1 patient as well as ERBB2 with RAF1, BRAF, and KRAS in 2 patients.

#### PI3KCA/AKT/mTOR pathway

**Fig 1:**
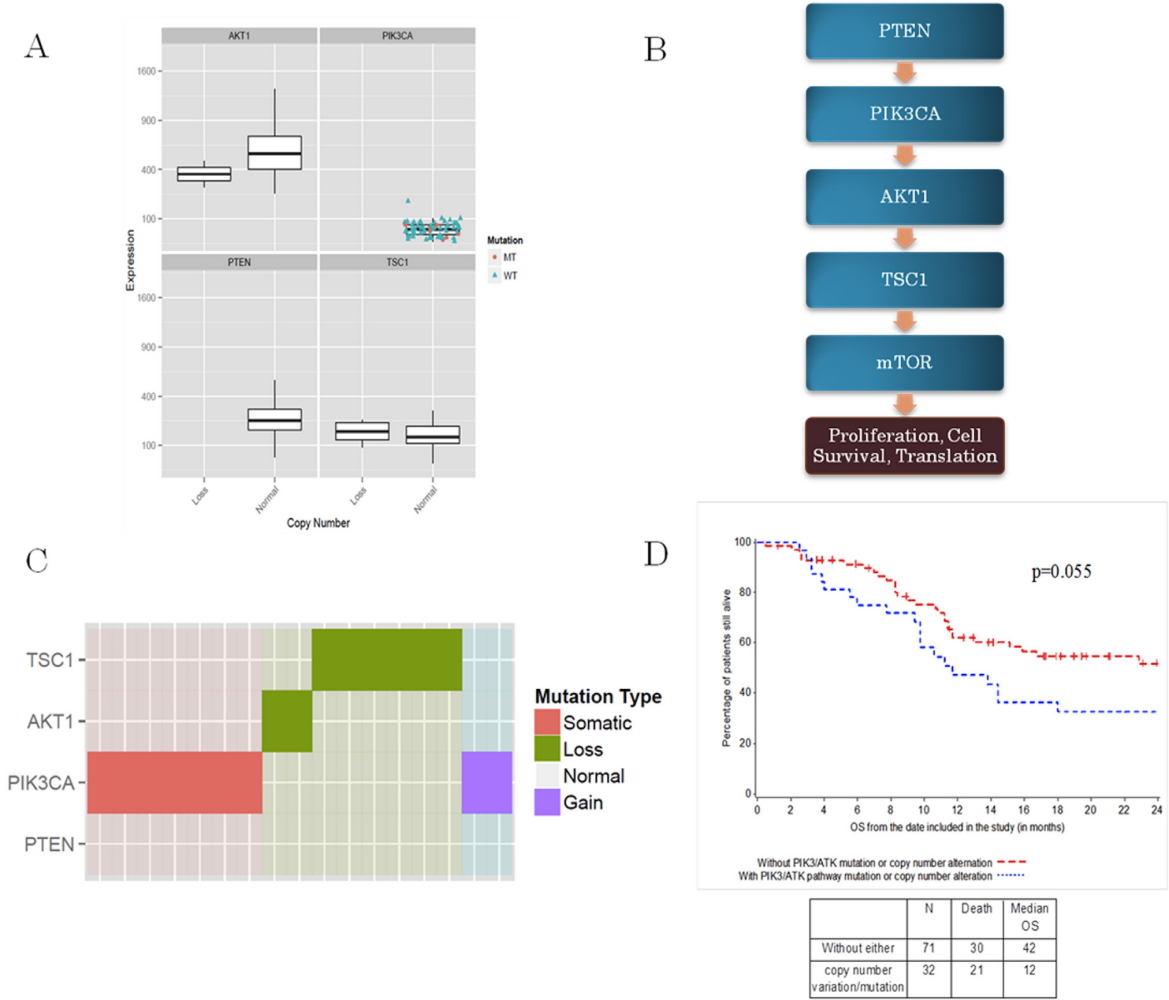
Association between mutations or CNV and OS. A: PIK3CA/AKT/mTOR pathway; B: Gene expression level analysis whithin the PI3KCA/AKT pathway; C: Heat map: mutations across the PIK3CA/AKT/mTOR pathway; D: Association between PIK3CA/AKT/mTOR pathway mutations or CNV and OS.

Thirty-two patients (31%) had genomic events within the PI3KCA/AKT signaling pathway: *PTEN* (copy number loss 6%), *PI3KCA* (copy number gain 10% or mutation 11%), *AKT1* (copy number gain 2%), *TSC1* (copy number loss, 13%), and *MTOR* (copy number gain 2%) (Fig 1). The only mutation observed within the PI3KCA/AKT pathways was PIK3CA (E545K and H1047R) (11%) (S1 Table) (Table 5).

**Table 5:** Association between gene expression levels and OS.

Gene Expression Level	N	Death	Median OS	p-value
***ERBB2***				0.85
≤709.86	40	18	18	
>709.86	39	19	16	
***TP53***				0.33
≤130.65	40	16	23	
>130.65	39	21	14	
***FGFR3***				0.52
≤170.95	40	20	14	
>170.95	39	17	23	
***KRAS***				0.10
≤293.99	40	15	Not reached	
>293.99	39	22	14	
***BRAF***				0.17
≤237.91	40	23	15	
>237.91	39	14	Not reached	
***RAF1***				0.26
≤143.65	40	16	25	
>143.65	39	21	16	
***PTEN***				0.08
≤214.00	40	14	Not reached	
>214.00	39	23	14	
***PIK3CA***				0.02
≤61.22	40	14	Not reached	
>61.22	39	23	14	
***AKT1***				0.70
≤497.23	40	18	23	
>497.23	39	19	16	
***TSC1***				0.68
≤138.41	40	20	14	
>138.41	39	17	18	
***CTNNB1***				0.0008
≤994.09	40	11	Not reached	
>994.09	39	26	12	
***TP53***				0.33
≤130.65	40	16	23	
>130.65	39	21	14	

Gene expression level analysis in the PI3KCA/AKT pathway was conducted. Expression levels were dichotomized at median. Overexpression of *PI3KCA* was found to be statistically significant correlated with OS (p = 0.02). Similarly, a trend of longer survival in patients with lower levels of *PTEN* gene expression was also observed (p = 0.08). We did not see significant associations between gene expression of any other genes in this pathway and OS (Table 3).

The association of CNV or mutation with OS within the whole PI3KCA/AKT pathway was very close to statistical significance (p = 0.055) (Fig 1). All of the genomic events within the PI3KCA were mutually exclusive. We then analyzed the association of PI3KCA pathway abnormality using CNV, mutation and gene expression data with OS. We were unable to show an association of these genomic events in any of the genes from PIK3/AKT pathway and OS (Table 6). When focusing at the gene level, *PI3KCA* abnormalities (CNV, mutation and gene expression) were associated with shorter OS (p = 0.04) (Table 7).

**Table 6:** Association between genomic events (CNV, mutation or gene expression) in any genes of PIK3/AKT pathway and OS.

	n	Death	Median OS	p-value
**PIK3/AKT pathway**				0.17
Without any abnormality	41	15	Not reached	
Copy number gain/mutation/high expression	38	22	14	

**Table 7:** Association of *PIK3CA* gene abnormality and OS.

	n	Death	Median OS	p-value
**PIK3CA gene**				0.04
Without any abnormality	34	11	Not reached	
Copy number gain/mutation/high expression	45	26	14	

#### WNT/CTNNB1 pathway

**Fig 2:**
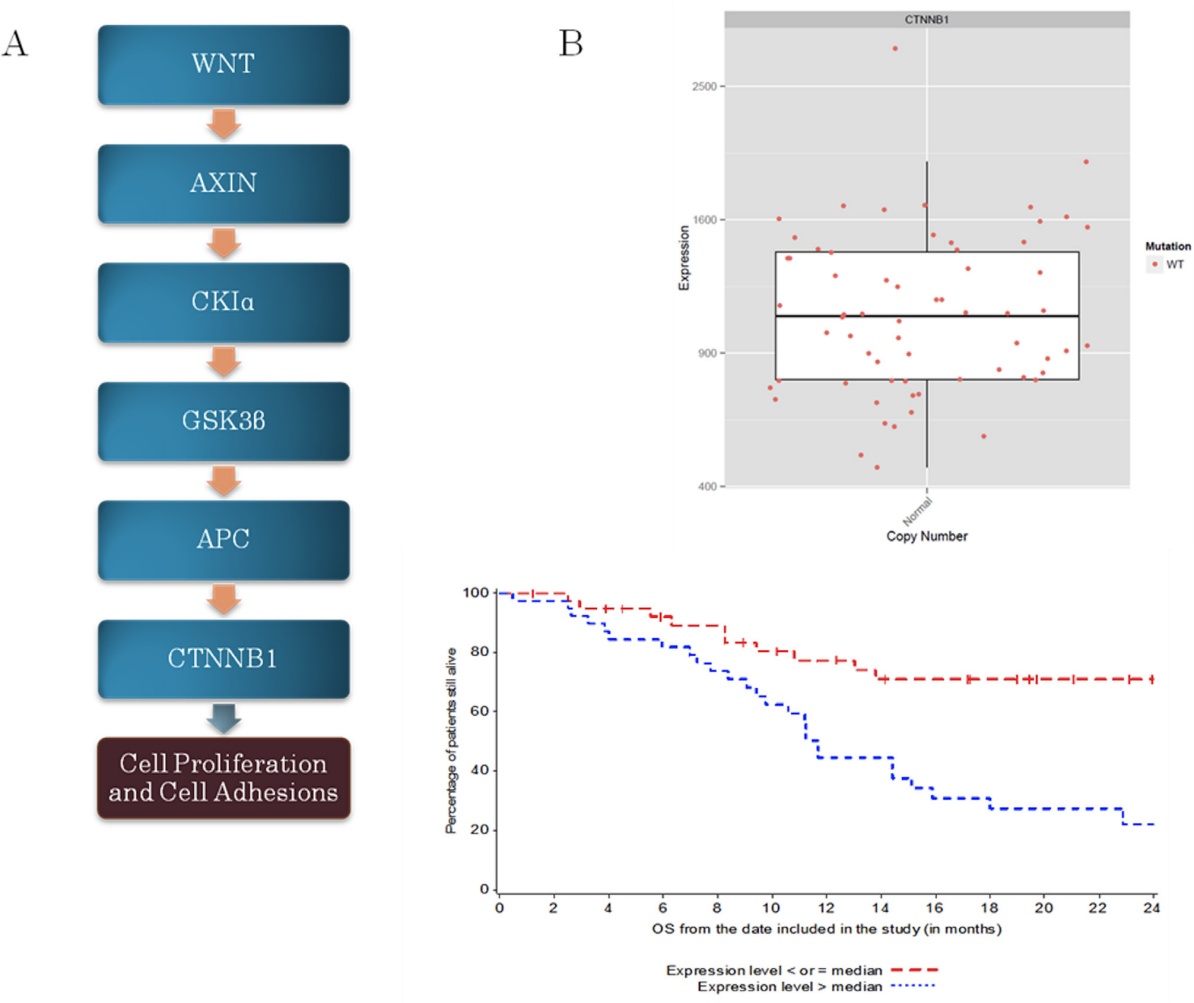
A: WNT/CTNNB1 pathway; B: Association between CTNNB1 overexpression and OS.

The WNT/CTNNB1 pathway includes *WNT*, Axin, APC, GSK3beta, CKIα, and *CTNNB1*. We observed one patient (1%) with *CTNNB1* mutation. When we examined the overexpression of *CTNNB1*, we found that CTNNB1 overexpression was associated with shorter OS (p = 0.0008) (Fig 2). No other genomic alterations were identified within this pathway.

#### RB1 Pathway

**Fig 3:**
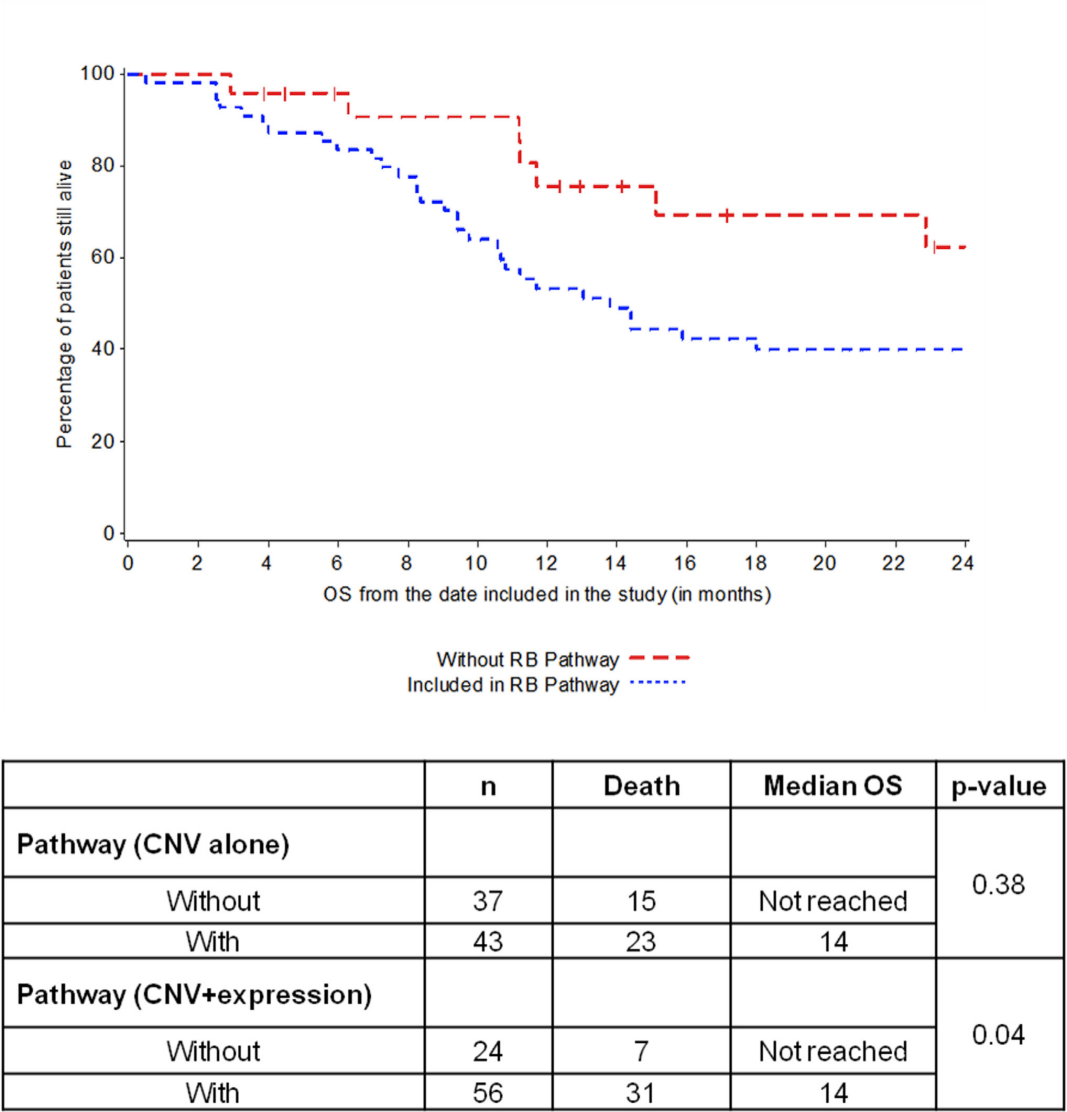
Association between RB1 pathway (CNV and expression) and OS.

Genomic events in the RB1 pathway were defined as *CCND1* copy number gain, *CCNE1* copy number gain, *CDKN2A* copy number loss, *E2F3* copy number gain, or *RB1* copy number gain. *CDK4* and *CDN2A* were not analyzed due to small number of patients with CNV. Gene expression level of each gene dichotomized at the median did not show any correlation with survival either alone or in combination with CNV. In an exploratory analysis, using the top or bottom 10% to categorize over- or underexpression, there was a statistically significant relationship favoring long-term survival in patients without RB1 pathway alteration (including CNV and gene expression) (p = 0.04) (Table 8; Fig 3).

**Table 8:** Association between genomic events in RB pathway (CNV with or without overexpression) and OS using 10% cutt-off.

	n	Death	Median OS	p-value
**Pathway (CNV alone)**				0.38
Without	37	15	Not reached	
With	43	23	14	
**Pathway (CNV+expression)**				0.04
Without	24	7	Not reached	
With	56	31	14	

## Discussion

We performed an integrative genomic analysis to determine the importance of individual genomic events in UC as well as the impact of those events when grouped into signaling pathways. The main finding in this study is that *PIK3CA* genomic events in the form of combined mutation, CNV and overexpression were statistically associated with shorter OS in patients with metastatic UC. A similar trend was observed at pathway level. We demonstrated that while there are many different genomic abnormalities within UC, there may be certain alterations which have a greater impact on survival. The correlation of genomic events with survival may help to better select targets for therapeutical intervention in UC[9,10].

Inactivating mutations or deletions in tumor suppressor genes like *PTEN* or *TSC1*, or amplifications or mutations in oncogenes like *PIK3CA* or *AKT1* have been reported to promote constitutive activation of PI3KCA/AKT/mTOR pathway in up to 40% of UC[11,12]. PI3KCA/AKT/mTOR pathway is involved in extracellular growth signaling, metabolism and cell proliferation and are potential therapeutic targets in advanced UC[13]. We observed a *PIK3CA* mutation rate of 11%, which is slightly under the 15–20% reported in the literature[14]. However, the presence of this mutation alone did not correlate with OS. The only individual genomic event in the PIK3CA pathway which correlated with a trend in OS benefit was a copy number gain of *mTOR* (p = 0.07).

Mutually exclusive patterns of alterations in the PI3KCA/AKT/mTOR pathway were identified in 31% of patients. Taken together, different genomic events (gene expression, mutation, or CNV) into this pathway correlated with shorter OS (p = 0.055). This could indicate that targeted therapies against the genes within the PI3KCA/AKT/mTOR pathway may lead to improved OS.

Loss or reduced expression of *PTEN* have been also associated with activation of PI3KCA/AKT/mTOR pathway supporting the rationale for better responses to mTOR inhibitors in patients with PTEN negative tumors[15,16]. In this analysis, we observed that overexpression of PTEN and PI3KCA were correlated with shorter OS (p = 0.08, p = 0.02 respectively). However *PI3KCA* mutations did not correlate with overexpression of PI3KCA.

Positive prognostic significance of *PIK3CA* mutations have been reported in different malignancies[17,18]. Recently, Kim and colleagues reported the results from a study in which next generation sequencing was performed to identify prognostic genomic biomarkers in high-grade UC[19]. As previously reported in other tumors, *PIK3CA* or PI3K/AKT/mTOR pathway abnormalities were associated with a statistically significant improvement in disease-specific outcome in patients with non-metastatic UC. Interestingly, the rate of recurrence was still high among these patients (44% in 5 years). In contrast, our findings suggest that in the metastatic setting abnormalities in this pathway may predict shorter OS. Given the tumor heterogeneity in UC, prospective studies investigating the prognostic significance in both, metastatic and non-metastatic setting, should be performed in larger cohorts. In addition, these findings provide a rational to investigate the impact of agents targeting PI3K/AKT/mTOR pathway in the adjuvant and metastatic setting in a population enriched by genomic alterations in this pathway.

*ERBB2* is reported to be amplified in 5% or more of tumor in UC[20–22]. In our study, copy number gains in *ERBB2* were identified in 19% of patients and were correlated with overexpression of the gene. However, neither overexpression nor CNV was correlated with OS. Although *ERBB2* overexpression has been correlated with survival in other tumor types, such as breast cancer, there has been no previous report of this correlation in UC[23]. Notably, two patients had mutation in *ERBB2*. The role of these mutations as potential drivers or targets in advanced UC is still under investigation.

Less than 15% of MIBC or metastatic UC tumors harbor *FGFR3* mutations[24]. In this analysis, 2 patients (2%) had activating mutation in *FGFR3*. It has been shown, in vivo and in vitro, that the inhibition of mutant *FGFR3* leads to cell cycle arrest and/or apoptosis[25], providing a rational for the development of FGFR3 inhibitors. Dovitinib, a small molecule FGFR3 and VEGFR inhibitor, has been tested in a phase II clinical trial and limited effectiveness was observed. The lack of activity in clinical studies could be related to inefficient targeting or inadequate testing[26]. Recent reports have shown that genetic translocations and rearrangements of *FGFR3* could activate the RKT/RAS/RAF pathway[27] and patients with these unusual alterations can be the population to respond to *FGFR3* inhibitors. These genomic alterations are now being investigated as potential therapeutic targets with next-generation FGFR3 inhibitors and are showing more promise[28,29] as seen recently in one of our patients included in a pan FGFR inhibitor clinical trial.

In our study, we observed a large number of CNV within the RB1 pathway, but no mutations in *RB1* gene were identified. We noted *RB1* mutations in about 15% of advanced UC and those mutations lead to greater CNV[30]. *RB1* mutations are rarely seen in low grade or low stage bladder tumors. Moreover, loss of heterozygosity at the RB locus (13q) is correlated with tumor grade and muscle invasion of bladder cancer[31]. The CNV variation seen in our analysis could indicate that *RB1* loss of heterozygosity could also induces genomic instability and promotes aneuploidy. The increase in genomic instability could indicate why *RB1* is correlated with higher grade and stage.

In addition, 12 patients (16%) had *TP53* inactivating mutations, which was less than the expected prevalence previously reported for metastatic UC of 34%[30]^30^. *TP53* plays essential roles in the regulation of cell proliferation, apoptosis and inhibition of angiogenesis. It is the most commonly mutated gene in cancer, including metastatic UC[7]. *TP53* mutation is correlated with survival, which is consistent with other metastatic bladder cancer literature. We observed a loss of *TP53* in 13% of patients. *TP53 loss* could be one reason we saw a lower prevalence of *TP53* mutations in our cohort. We did not see any mutations or copy number alterations in MDM2, an E3 ubiquitin protein ligase, which negatively regulates *TP53*.

Studies investigating *CTNNB1* mutations in UC have reported an average prevalence around 5%. In our cohort, only 1 patient (1%) had *CTNNB1* mutation. The WNT/CTNNB1 signaling pathway plays an important role in cell differentiation and tumorigenesis. Without a WNT signal, CTNNB1 is degraded, but when there is a WNT signal, CTNNB1 is allowed to accumulate and enter the nucleus, and regulate its target genes. A mutation on the *CTNNB1* allows it to escape the WNT regulation, which leads to an increase in transcription of its targeted genes[32]. In our study, *CTNNB1* overexpression was significantly associated with a shorter OS. Because CTNNB1 entering the nucleus depends on the accumulation of CTNNB1, overexpression of this protein would increase the transcription of target genes, leading to increased cell proliferation and subsequently poorer survival outcomes.

Despite the fact that this is the first study correlating pathway abnormalities in a homogeneous cohort of metastatic UC patients treated with platinum-based chemotherapy, several limitations need to be considered. First, the genomic analysis was done using a targeted sequencing platform (oncomap) that can limit the capture of existing mutations that could be sensitive to therapeutic intervention[33,34]. As an example, this platform does not identify the recently described high-frequency mutations affecting chromatin-modifying genes^37^ or potential downstream biomarkers as previously described[35]. Second, it is possible that the prevalence of genomic alterations differ between primary and metastatic tumors. An analysis of matched primary and metastatic samples from the same patient would address this possibility and should be a focus of future research efforts. Finally, there exists some degree of uncertainty regarding the tumor heterogeneity within our samples, as we sampled a single site. In our analysis, we saw genomic heterogeneity across the patient population, but it is possible that individual tumors were heterogeneous as well. Finally, given the small numbers of patients and low prevalence of genomic events, correction for multiple comparisons or exploratory analysis (i.e. correlation of genomic events and clinical parameters) may not result in reliable results, and thus these results are hypothesis generating and require further external validation.

In conclusion, this study plays an important role in deciphering the interdependent nature of cancer genomic alterations to guide targeted therapies. Our results suggest that targeted therapies focusing on the PI3KCA/AKT/mTOR pathway genomic alterations may improve clinical outcome in patients with metastatic urothelial cancer.

## Supporting Information

S1 Table Association of mutation status and OS. (PDF)
