# Supplementary material for: Somatic Copy Number Abnormalities and Mutations in PI3K/AKT/mTOR Pathway Have Prognostic Significance for Overall Survival in Platinum Treated Locally Advanced or Metastatic Urothelial Tumors
Source: PLoS One. 2015 Jun 3;10(6):e0124711. doi: 10.1371/journal.pone.0124711 (PMC4454515; doi:10.1371/journal.pone.0124711)
Supplement: S1 Table — (PDF) [file pone.0124711.s001.pdf]

Supplementary Table1: Association of mutation status and OS

	N	Death	Median OS	p-value
**PIK3CA**				0.26
Unmutated	77	41	14	
Mutated	9	4	Not reached	
**HRAS/KRAS**				0.17
Unmutated	81	40	17	
Mutated	4	4	12	
**TP53**				0.07
Unmutated	71	35	16	
Mutated	14	10	10	
